# Age of Insomnia Onset Correlates with a Reversal of Default Mode Network and Supplementary Motor Cortex Connectivity

**DOI:** 10.1155/2018/3678534

**Published:** 2018-04-01

**Authors:** Emiliano Santarnecchi, Chiara Del Bianco, Isabella Sicilia, Davide Momi, Giorgio Di Lorenzo, Salvatore Ferrone, Giulia Sprugnoli, Simone Rossi, Alessandro Rossi

**Affiliations:** ^1^Siena Brain Investigation & Neuromodulation Lab (Si-BIN Lab), Department of Medicine, Surgery and Neuroscience, Neurology and Clinical Neurophysiology Section, University of Siena, Siena, Italy; ^2^Berenson-Allen Center for Non-Invasive Brain Stimulation, Beth Israel Deaconess Medical Center, Harvard Medical School, Boston, MA, USA; ^3^Center for Sleep Medicine, “Le Scotte” Hospital, University of Siena, Siena, Italy; ^4^Department of Medicine, Surgery and Neuroscience, University of Siena School of Medicine, Siena, Italy; ^5^Laboratory of Psychophysiology, Chair of Psychiatry, Department of Systems Medicine, University of Rome “Tor Vergata”, Rome, Italy; ^6^Psychiatry and Clinical Psychology Unit, Department of Neurosciences, Fondazione Policlinico “Tor Vergata”, Rome, Italy

## Abstract

Insomnia might occur as result of increased cognitive and physiological arousal caused by acute or long acting stressors and associated cognitive rumination. This might lead to alterations in brain connectivity patterns as those captured by functional connectivity fMRI analysis, leading to potential insight about primary insomnia (PI) pathophysiology as well as the impact of long-term exposure to sleep deprivation. We investigated changes of voxel-wise connectivity patterns in a sample of 17 drug-naïve PI patients and 17 age-gender matched healthy controls, as well as the relationship between brain connectivity and age of onset, illness duration, and severity. Results showed a significant increase in resting-state functional connectivity of the bilateral visual cortex in PI patients, associated with decreased connectivity between the visual cortex and bilateral temporal pole. Regression with clinical scores originally unveiled a pattern of increased local connectivity as measured by intrinsic connectivity contrast (ICC), specifically resembling the default mode network (DMN). Additionally, age of onset was found to be correlated with the connectivity of supplementary motor area (SMA), and the strength of DMN←→SMA connectivity was significantly correlated with both age of onset (*R*^2^ = 41%) and disease duration (*R*^2^ = 21%). Chronic sleep deprivation, but most importantly early insomnia onset, seems to have a significant disruptive effect over the physiological negative correlation between DMN and SMA, a well-known fMRI marker of attention performance in humans. This suggests the need for more in-depth investigations on the prevention and treatment of connectivity changes and associated cognitive and psychological deficits in PI patients.

## 1. Introduction

Primary insomnia (PI) is a clinical condition characterized by troubles initiating or maintaining sleep, which is associated with daytime consequences and is not attributable to environmental circumstances or inadequate opportunity to sleep, as well as not to any other somatic or psychiatric cause [1]. In the last decades, PI has become more prevalent in industrialized nations (estimated to affect between 5% and 10% of the general population) [2, 3] and is associated with detrimental effects on cognition [4] as well as quality of life [5, 6], work productivity [7], and work-related injuries [8], as well as with increased vulnerability to general medical disorders [6, 9], psychiatric ones in particular [10, 11]. Recent neuroimaging studies have shed light on the potential neuroanatomical and functional correlates of PI, with structural magnetic resonance imaging (MRI) investigations showing abnormal grey matter volume in multiple brain regions, such as the hippocampus [12], medial frontal lobes [13], parietal cortex [14], and anterior cingulate cortex [15]. In addition to structural alteration, functional MRI (fMRI) studies have shown a variety of modifications induced by sleep deprivation or poor sleep quality, in both healthy participants [16] and patients with sleep disorders [17–20]. A variety of techniques and a priori hypotheses have been tested and validated, showing alterations of fMRI activity in regions related to attention and memory processing, as well as regions of the default mode network (DMN). However, these investigations, based on a priori selection of regions of interest (ROIs) capturing the activity of a given region/network [21], or based on an arbitrary anatomo-functional parcellation of the brain [22], might lead to a partial view of the possible insomnia-related alteration of brain activity, induced by sampling the activity of a limited subset of cortical and subcortical regions fitting with a given theory or pathophysiological model.

Moreover, recent evidence has suggested that insomnia might have an impact on both night and day brain functioning, with changes in brain plasticity, assessed via transcranial magnetic stimulation (TMS), reported in patients with PI [23]. Such a broad repercussion on central nervous system dynamics might also suggest an interaction between age-related brain plasticity mechanisms [24], length of exposure to sleep deprivation, and age of insomnia onset. Insight about how these factors might affect brain connectivity patterns, not available to date, might also help in defining novel therapeutic interventions and targets for insomnia.

Therefore, we investigated differences in resting-state functional connectivity fMRI patterns in drug-naïve patients with PI compared to healthy controls, looking at correlations between insomnia-induced alterations and clinical variable, in particular age of onset and disease duration. Importantly, in order to avoid a priori selection of analysis ROIs/masks, we implemented a high-resolution FC analysis based on voxel-wise connectivity maps indexing both local and distributed functional connectivity patterns for each voxel in the brain. We hypothesized that PI patients will display altered connectivity in sensory systems and/or regions related to attention and memory processing. We also hypothesized that insomnia duration and age of onset might exert similar effects on brain functional connectivity patterns, with early onset possibly leading to a stronger disruption of physiological brain dynamics.

## 2. Materials and Methods

### 2.1. Participants

Seventeen drug-naïve insomnia patients and 17 age- and education-matched healthy controls participated in the study. Diagnosis was based on the ICSD-3 criteria for primary insomnia (PI). All the participants were right-handed (as measured using the Oldfield handedness scale), cognitively intact (Mini-Mental State Examination score > 24), and monolingual native speakers and underwent a general physical and neurological screening, as well as an assessment of their medical history. PI patients were diagnosed and enrolled at the Center for Sleep Medicine of the Le Scotte Hospital (Siena, Italy). Each patient completed self-report clinical scales assessing the severity of their sleep-related complaints (Pittsburgh Sleep Quality Index (PSQI), Insomnia Severity Scale (ISI)) and their mood status (Beck Depression Inventory (BDI)). Inclusion criteria for patients were as follows: (1) fist diagnosis of primary insomnia at our center; (2) 18 to 45 years old; (3) no evidence of other medical disorders, with particular reference to current or past neurological and psychiatric ones or other sleep disorders; (4) no history of assumption of drugs acting on the central nervous system; and (5) no previous treatment or diagnosis of primary insomnia. They were advised to drink no more than one cup of coffee (or two of tea) and to not assume any amount of alcohol or other type of drink with caffeine in the day of the radiological examination. Healthy controls showed a normal neurological exam, regular sleep-wake cycle and no sleep complaints. Exclusion criteria were as follows: (1) abnormalities in physical and neurological examination screening visit, (2) current or past substance abuse, (3) use of psychotropic medication within 3 months prior to inclusion, and (4) brain structural abnormalities at the magnetic resonance imaging (MRI) exam. All participants gave their written informed consent to the experimental procedure, which conformed to the Declaration of Helsinki. The study was approved by the local ethical committee.

### 2.2. Clinical Assessment

Patients came to the Center for Sleep Medicine reporting sleep-related complaints involving “difficulty falling asleep or staying asleep, waking up early in the morning, and/or poor sleep quality with daytime consequences.” They were diagnosed for the first time by two neurologists (CDB and IS) licensed as Sleep Disorders Expert by the Italian Society for Sleep Medicine (Associazione Italiana Medicina del Sonno (AIMS); http://www.sonnomed.it/). The clinical evaluation included a clinical interview, assessment of clinical symptoms, evaluation of sleep diary, review of current and past medical and medication history, and clinical scales designed for depression (Beck Depression Inventory (BDI)), sleep disorders (Pittsburgh Sleep Quality Index (PSQI)), and insomnia in particular (Insomnia Severity Index). Patients had never assumed any drug treatment for insomnia, even if 3 patients have been trying herbal supplements until two months before undergoing the neuroradiological evaluation.

The Pittsburgh Sleep Quality Index (PSQI) [25] is a 19-item retrospective self-report questionnaire designed to provide a reliable, standardized measure of sleep quality and discriminate “good” and “poor” sleepers. Specifically, seven clinically derived domains are assessed (i.e., sleep quality, sleep latency, sleep duration, habitual sleep efficiency, sleep disturbances, use of sleep medications, and daytime dysfunction) composing a general score referring to global sleep quality. A score higher than 5 is considered an indicator of relevant sleep disturbances. A recently validated Italian version of the PSQI with high internal consistency was used [26].

The Insomnia Severity Index (ISI) is a brief self-report measure assessing perception of the severity of sleep disturbance [27]. Focusing on the past 1 month period, ISI evaluates the severity of sleep onset, sleep maintenance and early morning awakening problems, sleep dissatisfaction, interference of sleep difficulties with daytime functioning, noticeability of sleep problems by others, and distress caused by sleep difficulties. A 5-point Likert scale is used to rate each item (e.g., 0 = no complaint and 4 = severe complaint), with a total score ranging from 0 to 28. Higher scores indicate more severe insomnia, according to the following classifications: (i) absence of insomnia (0–7); (ii) subthreshold insomnia (8–14); (iii) clinical insomnia (moderate severity, 15–21); and (iv) clinical insomnia (severe, 22–28).

The Beck Depression Inventory (BDI) [28, 29] is a 21-item self-assessment questionnaire measuring the severity of symptoms and attitudes related to depression. It consists of 21 statements describing the somatic and cognitive-emotional symptoms of depression. Each item consists of four alternative responses graded from 0 to 3 according to the severity of the symptom. Patients are asked to choose the response better representing their mood state during the last 7 days. The total BDI score ranges from 0 to 63, with mild mood disturbance and clinical depression corresponding to, respectively, a score of 10 and 21 (or above).

### 2.3. MRI Data Acquisition

MRI data was acquired on a Philips Intera whole-body scanner. Resting-state fMRI data included 178 volumes with 33 axial slices covering the whole brain, acquired via a T2 BOLD-sensitive multislice echo planar imaging (EPI) sequence (TR/TE = 2.5 s/32 ms; field of view = 22 cm; image matrix =  64 × 64; voxel size =  3.44 × 3.44 × 3.8 mm^3^; flip angle = 75°). Structural imaging was performed using a whole brain T1-weighted Fast Field Echo 1 mm^3^ sequence (TR/TE = 30/4.6 ms, field of view = 250 mm, matrix 256 × 256, flip angle = 30°, slice number = 150, and scan time: 7 : 25 minutes). T2-weighted fluid-attenuated inverse recovery (FLAIR) images were also acquired to assess participants' white matter integrity. Participants were provided with earplugs and were instructed to lay in the scanner with their eyes open, while fixating on a cross hair. They were asked to stay as still as possible. To monitor the patients' state inside the scanner, the MRI technician monitored each patient via a camera placed inside the scanner for the entire fMRI acquisition. Particular care was taken to minimize head motion via vacuum cushions and custom-made padding.

### 2.4. fMRI Preprocessing

fMRI data preprocessing and statistical analyses were carried out using SPM8 software (Statistical Parametric Mapping; http://www.fil.ion.ucl.ac.uk/spm/), FSL for brain extraction procedure using the BET script (https://fsl.fmrib.ox.ac.uk/fsl/), and MATLAB 7.5 (MathWorks, MA, USA). The first three volumes of functional images were discarded for each subject to allow for steady-state magnetization. EPI images were slice-time corrected using the interleaved descending acquisition criteria and realigned and resliced using a mean functional volume derived from the overall fMRI scans. Subjects whose head motion exceeded 1.0 mm or rotation exceeded 1.0° during scanning were excluded. In order to obtain the better estimation of brain tissues maps, we implemented an optimized segmentation and normalization process using DARTEL (Diffeomorphic Anatomical Registration using Exponential Lie Algebra) [30] module for SPM8. Briefly, this approach is based on the creation of a customized anatomical template built directly from participants' T1-weighted images instead of the canonical one provided with SPM (MNI template, ICBM 152, Montreal Neurological Institute). This allows for a finer normalization into standard space and consequently avoids under- or overestimation of brain region volume possibly induced by the adoption of an external template. Hidden Markov Random Field model was applied in all segmentation processes in order to remove isolated voxels. Customized tissue prior images and T1-weighted template were smoothed using an 8 mm full-width at half-maximum (FWHM) isotropic Gaussian kernel. Functional images were consequently nonlinearly normalized to standard space, and a voxel resampling to isotropic 3 × 3 × 3 mm were applied. Linear trends were removed to reduce the influence of the rising temperature of the MRI scanner, and all functional volumes were band-pass filtered at (0.01 Hz < *f* < 0.08 Hz) to reduce the low-frequency drift. Finally, the CompCor algorithm has been applied in order to control physiological high-frequency respiratory and cardiac noise [31].

### 2.5. Intrinsic Connectivity Contrast

Individual connectivity maps were computed by means of the intrinsic connectivity contrast (ICC), a voxel-to-brain connectivity metric [32]. Differently from other local connectivity indexes, ICC takes into account not only the presence of a connection but also their strength, thereby producing voxel-based connectivity maps without the need for defining any ROIs. This index also has the advantage that it can be computed without applying a correlation threshold, and therefore, it does not require any a priori information or assumptions. ICC was applied according to the following formula:

(1) $$\begin{array}{c} ICC=\sum_{j}r{\left(i,j\right)}^{2}\cdot u\left(\left|r\left(i,j\right)\right|\right). \end{array}$$

ICC is computed for each voxel in the brain, therefore producing a whole-brain map where the intensity of each voxel reflects the average *R*^2^ connectivity of a given voxel *i* and all the other voxels in the brain. For statistical purposes, ICC values were normalized to fit a Gaussian distribution with zero mean and unitary variance by subtracting the ICC obtained at each voxel by the average value across all the voxels and dividing this by the standard deviation of the whole-brain map [33]. Resulting ICC maps have a spatial resolution of 3 mm^3^.

### 2.6. Functional Connectivity (FC) Analysis

Resting state FC analysis was implemented using ad hoc scripts implemented in a Python and MATLAB computational environment, based on code and modules from the same software used for preprocessing of MRI data. Analysis was based on voxel-wise connectivity indexes using the intrinsic connectivity contrast (ICC) [32] (see dedicated paragraph). To avoid any a priori hypothesis about specific brain regions or networks being involved in PI pathophysiology or correlated with symptoms, FC analysis was performed using a two-step procedure. (i) First, data were analyzed by comparing voxel-wise connectivity maps at the highest possible spatial resolution (3 mm^3^), looking for differences in resting-state (RS) brain activity at the single-voxel level. This provides a set of significant regions whose RS connectivity patterns are different (i.e., increased or decreased) between patients and controls, with this pattern representing either the activity of an isolated cluster of voxels with no clear anatomo-functional correspondence or actually matching the spatial topography of well-known resting-state networks [34]. This ensured that any result was not due to a priori selection of analysis masks or inflated by the reduction of statistical comparisons. However, even though significant clusters represent a spatially unconstrained information about “how” BOLD fMRI activity is different across groups or in relation to a given variable (e.g., age), they do not specify whether, for instance, the connectivity profile of cluster A (e.g., located in the right temporal lobe) is different in patients and controls because of its decreased connectivity with a specific other region of the brain, or multiple others, or an entire hemisphere, and so on. To derive such information, (ii) significant clusters were then introduced to a seed-based connectivity analysis investigating the pattern of connectivity between each significant voxel-level cluster and the rest of the brain. This two-step procedure allowed to obtain (i) unconstrained high-resolution targets not referring to any existing anatomo-functional atlas and (ii) a profile of their differences in connectivity as compared to healthy controls. Interestingly, according to the unconstrained nature of first-level analysis, the emergence of patterns of significant voxels resembling one or more known networks should be interpreted as a stronger indication of their relevance, given that no anatomical constraint was applied.

### 2.7. Statistical Analysis

#### 2.7.1. Group Differences

Voxel-wise connectivity maps were compared across PI patients and HC, using an analysis of covariance (ANCOVA) including age, gender, and BDI score as covariates. Results were considered significant at a threshold equal to *p* < 0.05, with false discovery rate (FDR) correction. As specified above, significant clusters were then used as seed regions in a second-level seed-based connectivity analysis. The same statistical thresholds were applied in both analyses.

#### 2.7.2. Correlation with Clinical Scores

The same approach was used to derive patterns of disease-related modifications in patients' connectivity profile. Analysis were run only in PI patients (*n* = 17). Voxel-wise regression models were built by predicting age of onset, disease duration, and ISI scores (FDR, *p* < 0.05; FWE, *p* < 0.05), followed by seed-based analysis using resulting significant clusters.

## 3. Results

### 3.1. Clinical Assessment

The selected PI patients (*n* = 17) reported an average age of onset of 28.82 ± 10.15 yrs, with an average score at the PSQI of 15 ± 2.35 and an ISI of 18.45 ± 5.16. They also did not report a significant deflection in mood levels (BDI = 8 ± 4.52) and no clinically significant cognitive decline (MMSE = 28 ± 2). Healthy controls also did not report any mood-related symptomatology (BDI = 6 ± 3.86; group comparison: *t* = 1.3873, *p* = 0.275) and an intact cognitive profile (MMSE = 29 ± 1; group comparison: *t* = 1.8439, *p* = 0.115).

### 3.2. Voxel-Wise Connectivity Group Differences

**Figure 1:**
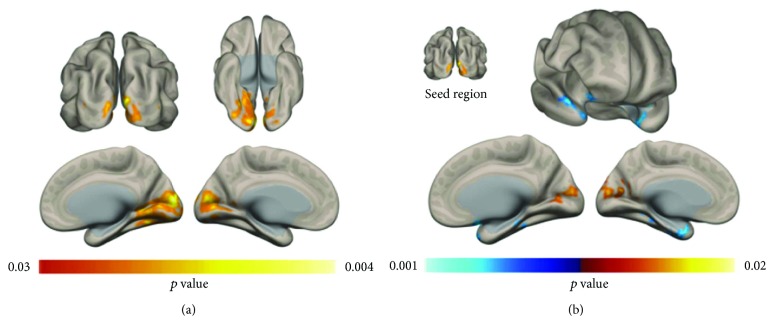
Voxel-wise FC changes. An increase in resting-state voxel-to-brain connectivity was observed in the occipital lobe of PI patients with respect to healthy controls. The seed-based connectivity profile of the significant occipital lobe cluster in (a) was compared across groups, highlighting an increase in local connectivity in PI patients and a decrease in connectivity in the bilateral temporal pole (b) (*p* < 0.05; FDR corrected, FWE corrected cluster-level).

Analysis of voxel-wise connectivity maps lead to significant differences in ICC patterns. Increase in connectivity of the bilateral occipital lobe was observed in PI patients with respect to controls (Figure 1(a)). Subsequent seed-based analysis highlighted a pattern of increased connectivity between bilateral occipital lobe and superior occipital lobe (i.e., increased local connectivity), as well as a decrease in connectivity with bilateral temporal pole structures (Figure 1(b)). Statistical results and anatomical localization of each cluster are reported in Table 1.

### 3.3. Correlation with Clinical Scores

**Figure 2:**
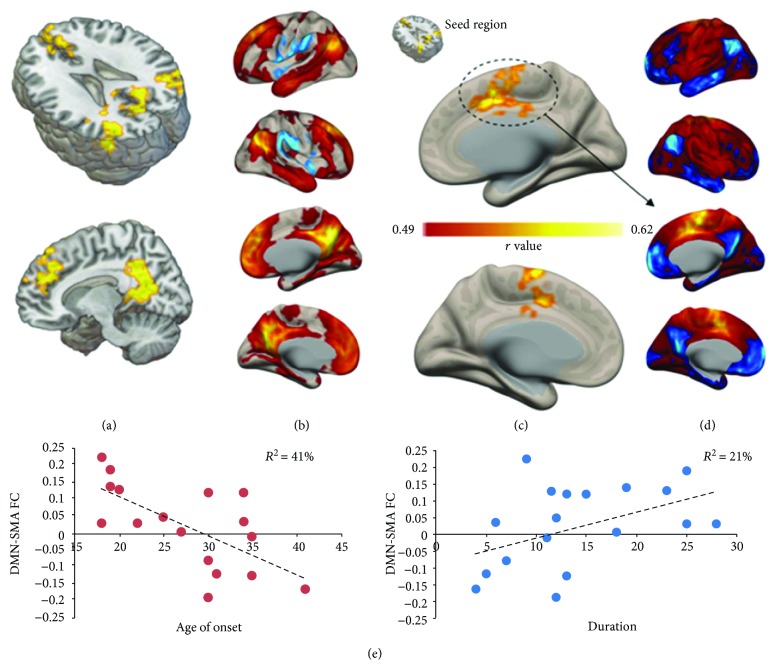
Correlation with clinical scores. Results of the voxel-wise correlation between FC patterns and age of onset are shown in (a), highlighting a set of clusters closely resembling the default mode network (DMN), as confirmed by the seed-based connectivity profile of the same clusters computed on the entire study sample (b). Specifically, age of onset was positively correlated with the connectivity between the DMN cluster in (a) and the bilateral supplementary motor area (SMA) (c). By looking at resting-state activity in PI patients (c), SMA displays a negative correlation with the DMN in healthy controls (d), suggesting that chronic sleep deprivation might weaken such resting-state dynamic. Scatterplots (e) display individual FC strength between the DMN clusters in (a) and SMA in (c) as a function of age of onset and disease duration (*p* < 0.05; FDR corrected, FWE corrected cluster-level), corroborating such hypothesis by showing how early onset and longer insomnia duration correspond to a modification of DMN-SMA connectivity in PI patients (i.e., a reversal in patients with early onset and longer insomnia duration, from negative to positive connectivity).

The regression model predicting age of onset highlighted a pattern of increased ICC in multiple clusters of voxels resembling the default mode network (DMN; Figures 2(a) and 2(b); Table 2). In particular, seed-based analysis unveiled a significant correlation between the DMN cluster shown in (a) and (b) and the bilateral supplementary motor area (SMA; Figure 2(c)), a brain region that is negatively correlated with the DMN in healthy controls (Figure 2(d)). No significant patterns were observed for insomnia severity (*p* = 0.21), whereas close to significance results were obtained for disease duration (*p* = 0.08). Statistical results and anatomical localization are reported in Table 2. Correlation between the strength of DMN-SMA connectivity and clinical scores are displayed in Figure 2(e), accounting for 21% and 41% of variance in insomnia duration and age of onset, respectively.

## 4. Discussion

Data showed how chronic insomnia is able to induce changes in brain connectivity, with a specific impact on visual cortex resting-state activity. Moreover, individual differences in age of onset and insomnia duration were identified as a predictor of changes of connectivity patterns between the DMN and a core region of the motor system (SMA). Interestingly, age of onset displayed a significantly stronger correlation with fMRI alteration than disease duration, suggesting the importance of addressing insomnia-related effects on brain connectivity in younger adults to prevent long-lasting connectivity reshaping.

### 4.1. Insomnia-Induced Changes in FC

The most prominent difference in voxel-wise FC between PI patients and healthy controls was evident in the bilateral visual cortex. Interestingly, this finding has not been reported in any previous fMRI study on PI, whereas it fits with prior evidence of abnormal FC pattern within the occipital cortex in sleep-deprived healthy subjects [21]. Also, Morgan and colleagues reported a selectively increased occipital *γ*-aminobutyric acid (GABA) level in PI patients as compared to healthy controls [35]. A similar result, but extended to the entire brain, was reported by Nofzinger and colleagues, showing a greater global cerebral glucose metabolism during NON-REM sleep and wakefulness in patients with insomnia [36]. The occipital hyperactivation observed in PI patients fits with the hyperarousal theory of insomnia [37], positing a hypersensibility to external stimuli which might be driven by an overactivity of visual brain regions in patients. Increased connectivity within visual, and other sensory regions, may contribute to sustained sensory processing of environmental stimuli, ultimately hampering the ability to initiate or maintain sleep [38]. This also fits with a recent evidence of increased connectivity between the insula and the salience networks in patients with PI [39], given the role of regions of the salience network in, among other functions, monitoring bodily sensation and attribute salience to external and proprioceptive stimuli.

In addition, seed-based analysis highlighted a reduction of connectivity between the occipital lobe and two clusters mapping on the bilateral temporal pole, in particular with the hippocampus. While modifications of temporal lobe activity have been reported in PI patients [12], to our knowledge, this specific occipito-temporal connectivity change is novel. The impact of sleep deprivation on temporal lobe structures is widely documented in both humans and animal models, with disruption of memory consolidation processes [40] and local connectivity patterns [41]. Interestingly, human anatomo-functional data reported the connection between occipital and temporal cortex as part of a network involved in processing of visual stimuli [42, 43], a process possibly facilitated by the presence of a direct white matter fiber bundle connecting the hippocampal region and the visual cortex (inferior longitudinal fasciculus) [44]. Our results might point to changes in consolidation of visually encoded information, suggesting that the hyperactivity of visual brain regions in PI patients could cause the observed occipito-temporal functional “disconnection,” presumably having a “protective” effect on temporal pole function. Future investigations are needed to understand whether such alteration has a specific clinical meaning or just represents a wider and less specific set of connectivity changes resulting from chronic sleep deprivation.

### 4.2. Correlation with Insomnia Duration and Age of Onset

A significant correlation between individual connectivity patterns and both insomnia duration and age of onset was also found. We highlighted a very interesting correlation, yet preliminary and limited by sample size, between the age of onset and the strength of connectivity of regions highly resembling the DMN (i.e., medial prefrontal cortex, precuneus, and bilateral angular gyrus). Interestingly, using resting-state fMRI in healthy controls under controlled sleep deprivation, two studies have demonstrated an aberrant functional activity both within the DMN and between the DMN and its negatively correlated regions [16, 45]. On the other hand, greater sleep time the night before the fMRI scan seems to correlate with increased RS connectivity between two nodes of the DMN (medial prefrontal cortex and posterior cingulate cortex) in healthy volunteers, as well as with more negative correlations between these regions and those parts of negatively correlated resting-state networks (lateral prefrontal regions, parietal attention, and occipital sensory cortices) [46]. Furthermore, a longitudinal structural MRI study has also recently documented a structural disconnection between anterior and posterior regions of the DMN in patients with PI compared to healthy controls [47]. All these results point to an insomnia-related alteration of DMN activity, a network known for his role in memory processing as well as in attention-related processes when its negative correlation with other networks is considered [48, 49]. Interestingly, seed-based analysis originally highlighted the source of DMN's activity alteration in an increased positive connectivity with bilateral SMA. Specifically, patients with early age of onset (i.e., around 20–25 years old) display a reversal of resting-state DMN-SMA connectivity patterns, that is, a positive connectivity instead of the widely reported negative correlation between DMN fMRI oscillatory activity and that of the rest of the brain [48]. The involvement of a motor system region as the SMA in PI patients might be surprising, but evidence involving motor system alterations in both patients and healthy controls have been reported. For instance, a recent EEG study demonstrated significantly elevated spectral power values in the EEG beta frequency band during NREM stage 2 in PI patients, an EEG feature mainly viewed as a general index of cortical arousal in sleep [50]. Even more interestingly, a recent study has documented the specific role played by DMN-SMA connectivity during a vigilance/attention task in healthy controls [51]. The authors reported how better individual performance at the task performed in the MRI scanner specifically reflects the strength of the negative correlation between DMN and SMA. The presence of a strong negative correlation corresponded to shorter reaction times and better overall performance, while a reversal of such dynamic, captured by looking at second-by-second BOLD fMRI activity, leads to a general worsening of attention. The reduced DMN-SMA negative correlation observed in our sample might represent one of the neurofunctional substrates of patients' attention deficits and should be explored with ad hoc experimental designs.

Age of onset-related remapping of brain functional architecture might be related to plasticity mechanisms, which seems to change across the lifespan [24] and to be amplified in the younger brain [52]. A recent report has suggested that PI patients have altered use-dependent plasticity (UDP), one of the mechanisms underlying formation of motor memory traces and considered a sensitive measure to assess neuroplasticity in the motor system as well as a proxy of brain plasticity in general. By using transcranial magnetic stimulation (TMS), the authors found that insomnia patients display increased UDP changes relative to controls, also showing enhanced intracortical facilitation (i.e., an index of glutamatergic mechanism) relative to controls, in the absence of changes in intracortical inhibitory (GABAergic mechanism) measures [23]. Overall, patients seemed to show a heightened state of neuroplasticity possibly due to altered glutamatergic circuits and reflecting a form of maladaptive plasticity. A similar mechanism might be responsible for the observed modulation of functional connectivity depending on age of onset, suggesting the need for longitudinal TMS-based assessment of cortical plasticity in patients across the lifespan and age-of-onset distribution.

If replicated in independent samples, the reversal of DMN-SMA dynamics highlighted in PI patients will suggest the need of early interventions aimed at counteracting such disruption of resting-state brain connectivity patterns, possibly using noninvasive brain stimulation (NIBS) [53, 54] techniques. The use of NIBS in sleep disorders has not been extensively explored [55], with the recent technical evidence of the possibility of targeting specific fMRI networks, instead of single brain regions, possibly representing an intuitive approach to engage the DMN and preserve its physiological negative correlation. Moreover, insomnia has a high comorbidity with depression, and the two conditions share some of the neurobiological markers, making the quest for identifying stimulation targets even more important. Most importantly, PI is no longer considered a secondary condition to depression but rather an independent clinical entity; while insomnia is a risk factor for depression onset [56, 57], depression treatments are not a sufficient remedy for insomnia [58]. Even further, it seems that insomnia-targeted cognitive behavioral therapy (CBT) might be a better therapeutic approach to cure both insomnia and depression than CBT based on depression symptoms: a randomized trial comparing the efficacy of CBT for insomnia and depression (tested in separate groups of patients with both diagnoses) have shown insomnia treatment inducing more beneficial effects than depression treatment, in both conditions [59]. Combining CBT and network-based brain electrical stimulation might be an option to be considered. Also, the need for a better understanding of insomnia pathophysiology and possible restorative options is even more important when considered in the context of neurodegenerative disorders, with the recently documented impact of sleep-deprivation on amyloid clearance [60], the link between sleep-wake cycle and amyloid dynamics [61], as well as the general association between sleep deprivation and age of Alzheimer's disease onset.

### 4.3. Conclusion

The present findings suggest the importance of exploring the role of brain plasticity mechanism into compensating for early insomnia onset and prolonged exposure to sleep deprivation. Functional data also suggest a significant enhancement of resting-state activity in the visual cortex of PI patients, corroborating the hyperarousal theory of insomnia and possibly representing a target for therapeutic interventions.

## Figures and Tables

**Table 1:** Group differences in functional connectivity. Localization of the voxel-wise connectivity differences between PI patients and healthy controls are reported, with corresponding MNI coordinates. The results of seed-based analysis between the ICC cluster and the rest of the brain are also shown.

Procedure	Cluster MNI coordinates			*k*	Cluster localization	Cluster p-FDR	Increased/decreased connectivity
	*x*	*y*	*z*				
*ICC—voxel wise*	14	−90	4	2328	973 voxels, primary visual cortex (left)	0.00005	↑
					1076 voxels, primary visual cortex (right)		
					341 voxels, lingual gyrus (right)		
					132 voxels, lingual gyrus (left)		
*Seed-based*	38	−66	12	2893	477 voxels, brain stem	0.00003	↓
					153 voxels, frontal orbital cortex (right)		
					139 voxels, hippocampus (right)		
					132 voxels, temporal pole (right)		
					111 voxels, lateral occipital cortex, inferior division (right)		
	−42	−48	0	1702	117 voxels, middle temporal gyrus, posterior division (left)	0.0002	↓
					112 voxels, amygdala (left)		
					92 voxels, hippocampus (left)		
					80 voxels, parahippocampal gyrus, anterior division (left)		
					51 voxels, middle temporal gyrus, temporooccipital part (left)		
	0	−86	10	590	156 voxels, intracalcarine cortex (left)	0.008556	↑
					96 voxels, intracalcarine cortex (right)		

**Table 2:** Correlation with age of onset. Results for both voxel-wise ICC and seed-based analysis are reported, with corresponding cluster size and coordinates in MNI space.

Procedure	Cluster MNI coordinates			*k*	Cluster localization	Cluster p-FDR	Increased/decreased connectivity
	*x*	*y*	*z*				
*ICC—voxel wise*	8	38	28	5609	759 voxels, paracingulate gyrus (right)	0.00003	↑
					758 voxels, superior frontal gyrus (right)		
					480 voxels, superior frontal gyrus (left)		
					456 voxels, paracingulate gyrus (left)		
					439 voxels, frontal pole (left)		
					261 voxels, middle frontal gyrus (right)		
					255 voxels, cingulate gyrus, anterior division		
					243 voxels, frontal medial cortex		
					125 voxels, middle frontal gyrus (left)		
	8	−56	38	5103	2996 voxels, precuneus cortex	0.00004	↑
					1249 voxels, cingulate gyrus, posterior division		
					122 voxels, cuneal cortex (right)		
					107 voxels, intracalcarine cortex (right)		
	44	−68	26	1602	1220 voxels, lateral occipital cortex, superior division (right)	0.000006	↑
					303 voxels, angular gyrus (right)		
	−44	−62	24	1264	1000 voxels, lateral occipital cortex, superior division (left)	0.00007	↑
					228 voxels, angular gyrus (left)		
*Seed-based*	2	16	56	3671	1790 voxels, supplementary motor area (left)	0.0002	↓
					1540 voxels, supplementary motor area (right)		
